# QsdH, a Novel AHL Lactonase in the RND-Type Inner Membrane of Marine *Pseudoalteromonas byunsanensis* Strain 1A01261

**DOI:** 10.1371/journal.pone.0046587

**Published:** 2012-10-08

**Authors:** Wei Huang, Yongjun Lin, Shuyuan Yi, Pengfu Liu, Jie Shen, Zongze Shao, Ziduo Liu

**Affiliations:** 1 State Key Laboratory of Agricultural Microbiology, College of Life Science and Technology, Huazhong Agricultural University, Wuhan, P.R. China; 2 The State Oceanic Administration, The Third Marine Research Institute, Xiamen, P.R. China; 3 National Key Laboratory of Crop Genetic Improvement, College of Life Science and Technology, Huazhong Agricultural University, Wuhan, P.R. China; Centre National de la Recherche Scientifique, Aix-Marseille Université, France

## Abstract

*N*-acyl-homoserine lactones (AHLs) are the main quorum-sensing (QS) signals in gram-negative bacteria. AHLs trigger the expression of genes for particular biological functions when their density reaches a threshold. In this study, we identified and cloned the *qsdH* gene by screening a genomic library of *Pseudoalteromonas byunsanensis* strain 1A01261, which has AHL-degrading activity. The *qsdH* gene encoded a GDSL hydrolase found to be located in the N-terminus of a multidrug efflux transporter protein of the resistance-nodulation-cell division (RND) family. We further confirmed that the GDSL hydrolase, QsdH, exhibited similar AHL-degrading activity to the full-length ORF protein. QsdH was expressed and purified to process the N-terminal signal peptide yielding a 27-kDa mature protein. QsdH was capable of inactivating AHLs with an acyl chain ranging from C_4_ to C_14_ with or without 3-oxo substitution. High-performance liquid chromatography (HPLC) and electrospray ionization-mass spectrometry (ESI-MS) analyses showed that QsdH functioned as an AHL lactonase to hydrolyze the ester bond of the homoserine lactone ring of AHLs. In addition, site-directed mutagenesis demonstrated that QsdH contained oxyanion holes (Ser-Gly-Asn) in conserved blocks (I, II, and III), which had important roles in its AHL-degrading activity. Furthermore, the lactonase activity of QsdH was slightly promoted by several divalent ions. Using *in silico* prediction, we concluded that QsdH was located at the first periplasmic loop of the multidrug efflux transporter protein, which is essential to substrate selectivity for these efflux pumps. These findings led us to assume that the QsdH lactonase and C-terminal efflux pump might be effective in quenching QS of the *P. byunsanensis* strain 1A01261. Moreover, it was observed that recombinant *Escherichia coli* producing QsdH proteins attenuated the plant pathogenicity of *Erwinia carotovora*, which might have potential to control of gram-negative pathogenic bacteria.

## Introduction

Numerous bacteria monitor their own population densities by sensing the concentration of small signaling molecules called autoinducers, which further regulate the expression of specific genes, thus resulting in better adaptation to a novel environment [1]. The cell-to-cell communication phenomenon of quorum sensing (QS) depends on the production, secretion and response to diffusible autoinducers [1]. Many gram-negative bacteria communicate with each other through AHLs. Once these chemicals reach a threshold concentration, they activate QS-dependent gene expression and produce phenotypic effects, including bioluminescence, Ti plasmid conjugal transfer and swarming motility [2], [3]. Many pathogens rely on quorum sensing to synchronize microbial activities essential for infection by triggering expression of particular virulence genes [4], [5]. Recently, many quorum-quenching phenomena have been observed, and strategies have been developed to disturb quorum sensing. The discovery of quorum-quenching mechanisms has demonstrated that these mechanisms are widely conserved in many prokaryotic and eukaryotic organisms and have important roles in microbe/microbe and pathogen/host interactions.

To date, several groups of potent quorum-quenching enzymes have been identified, including AHL lactonase, AHL amidohydrolase (AHL acylase), paraoxonases (PONs), and AHL oxidoreductase [4], [6]. AHL lactonases catalyze the lactone ring opening by hydrolyzing AHL. The first cloned AHL lactonase was *aiiA* from *Bacillus* spp. 240B1 (hereafter referred to as AiiA_240B1_) [7] followed by *aiiB* and *attM* from *Agrobacterium* [8], [9], *ahlD* from *Arthrobacter* spp. [10], *qsdA* from *Rhodococcus erythropolis* strain W2 [11], *aiiM* from *Microbacterium testaceum* StLB037 [12], and *aidH* from *Oclrobacterium* spp. [13]. These AHL lactonases come from several different hydrolase families, such as the metallo-beta-lactamase superfamily, α/β hydrolase superfamily, and PTE superfamily. Site-directed mutagenesis based on a sequence alignment of AiiA homologues has elucidated that AiiA contains a HXHXDH zinc-binding motif, which is widely conserved in several groups of metallohydrolases and is essential for the enzymatic activity of AHL lactonase [7]. In contrast with previous studied AHL lactonases, AiiM and AidH show similarity to predicted α/β hydrolase fold family proteins but exhibit variable substrate specificity of AHLs [12], [13]. Similarly, several bacteria have been reported to encode an AHL acylase for hydrolyzing the amide bond of AHL and releasing the fatty acid. For example, AHL acylases have been discovered in *Ralstonia* spp. XJ12B, *Streptomyces* spp. M664, *Shewanella* spp., *Pseudomonas aeruginosa*, *Ralstonia solanacearum* GM1000, and *Ochrobactrum* sp. A44 [3], [14], [15], [16], [17], [18], [19], [20]. Interestingly, strong AHL inactivation activity of paraoxonases has been observed in mammalian species. The human paraoxonase gene family has three members (PON1, PON2 and PON3) that exhibit wide physiological effects, including quorum-quenching enzymatic activity. Enzymatic characterization of purified PONs has revealed them to be lactonases with some overlapping substrates (e.g., aromatic lactones) [21], [22]. *In vitro* assays have shown that PON1 from mouse serum is required and sufficient to degrade 3OC12-HSL and that PON2 and PON3 also effectively degrade 3OC12HSL [22].

All AHLs are amphipathic molecules that have been assumed to be freely diffuse or to be transported outside of bacterial cells [1]. Recently, studies have found that short-chain AHL signals can freely diffuse across bacterial membrane but that long-chain AHLs are not freely permeable. In *Pseudomonas aeruginosa*, cell-to-cell signaling controls the expression of extracellular virulence factors and biofilm differentiation [23]. To date, several multidrug efflux systems have been described in *P. aeruginosa* that contribute to the active efflux of AHLs, *N*-butanoyl-L-homoserine lactone (C4HSL) and *N*-(3-oxododecanoyl) homoserine lactone (3OC12HSL) and then regulate the intracellular signal molecule levels. For example, 3OC12HSL efflux in *P. aeruginosa* cells relies on active transportation by the MexAB-OprM multidrug efflux pump [24]. Overexpression of MexEF-OprN is correlated with a decrease in the production of extracellular virulence factors, particularly those controlled by the *rhl* system, thus affecting the intracellular C4HSL and *Pseudomonas* quinolone signal (PQS) [25]. Similarly, the MexGHI-OpmD pump in *P. aeruginosa* cells involves an inability to produce 3OC12HSL and PQS as well as a drastic reduction of *N*-butanoyl-L-homoserine lactone levels, thus indicating its essential function in facilitating cell-to-cell communication and antibiotic susceptibility in addition to promoting virulence and growth [26]. In *Burkholderia pseudomallei,* the extracellular secretion of AHL relies on the BpeAB-OprB efflux pump [27], [28], which is required for efflux but not for influx of acyl-HSLs during quorum sensing [27]. These active transportation mechanisms reduced the production of AHL signals and virulence factors.

In this study, we reported the isolation of the AHL-inactivating enzyme, QsdH, which is a GDSL hydrolase from *P. byunsanensis* strain 1A01261. QsdH was found to be located in the N-terminus of a multidrug efflux transporter protein functioning as a novel broad spectrum AHL lactonase. In addition, QsdH was found to be integrated into the C-terminal cytoplasmic RND-type membrane protein, which is the first known example of a multidrug efflux pump transporter protein with an N-terminal periplasmic extension in the genus *Pseudoalteromonas.* Additionally, the recombinant protein may regulate the expression of certain phenotypic genes by controlling intercellular AHL levels in *P. byunsanensis* 1A01261. The result showed that over-producing QsdH in *E. coli* inhibits virulence of *E. carotovora*, a pathogenic bacterium of plants.

## Materials and Methods

### Chemicals, strains, plasmids and culture conditions

*N*-butanoyl-L-homoserine lactone (C4HSL), *N*-nexanoyl-L-homoserine lactone (C6HSL), 3-oxohexanoyl-L-homoserine lactone (3OC6HSL), *N*-octanoyl-L-homoserine lactone (C8HSL), 3-oxooctanoyl-L-homoserine lactone (3OC8HSL), *N*-decanoyl-L-homoserine (C10HSL), *N*-dodecanoyl-L-homoserine (C12HSL) and *N*-tetradecanoyl-L-homoserine lactone (C14HSL) were purchased from the Cayman Chemical Company. All strains and plasmids used in this study are listed in Table 1. *E. coli* were cultured in Luria-Bertani medium (the pH was buffered to 6.8 using HCl) at 37°C. The marine bacteria were supplied by the Marine Culture Collection of China, and were cultured in modified LB medium (1% tryptone, 3% NaCl, and 0.5% yeast extract; pH 7.0) at 25°C. The *Agrobacterium tumefaciens* strain NT1 (*traR*; *tra*::*lacZ749*) and *E. carotovora* SCG1 was cultured in a minimal medium as previously described (Zhang et al, 2000). For a bioassay plate to measure AHL degradation, 5-bromo-4-chloro-3-indolyl-β-D-galactopyranoside (X-gal) was added to minimal solid medium to a final concentration of 40 µg/ml. In addition, ampicillin was added to a final concentration of 100 µg/ml.

**Table 1:** Bacterial strains and plasmids used in the study.

Strain or plasmid	Characteristics	source and reference
**Strains**		
*E. coli* strain DH5α	a host for DNA cloning	Lab collection
*E. coli* BL21 (DE3)	a cloning and expression host	Lab collection
*E. carotovora* SCG1	plant-pathogenic bacteria	Lab collection
*A. tumefaciens* NT1	AHL-detecting strain; *traR*; *tra*::*lacZ749*	Dr. Lianhui Zhang
*P. byunsanensis* 1A01261	AHL-degrading strain; marine bacteria	MCCC
**Plasmids**		
pUC118	Ap^r^	Lab collection
pUC118-*sm20*	Ap^r^; screened target gene inserted into pUC118	In this study
pUC118-*orf*	*orf* amplified and cloned into pUC118	In this study
pUC118-*orf'*	*orf* (process signal peptide) amplified and cloned into pUC118	In this study
pUC118-*qsdH*	GDSL hydrolase gene *qsdH* amplified and cloned into pUC118	In this study
pGEX-6p-1	Ap^r^	Lab collection
pGEX-6p-*qsdH*	GDSL hydrolase gene *qsdH* amplified and cloned into pGEX-6p-1	In this study

MCCC, Marine Culture Collection of China.

### Screening of AHL-degrading marine bacteria

The biosensor *A. tumefaciens* strain NT1 was applied to detect residual AHLs on agar plates as previously described [7], [29]. Strains from the Marine Culture Collection of China (MCCC) were cultured at 28°C with gentle shaking to an OD_600_ of approximately 1.5. The cells were collected by centrifugation, washed and suspended in 200 µl of PBS buffer (140 mM NaCl, 2.7 mM KCl, 10 mM Na_2_HPO_4_ and 1.8 mM KH_2_PO_4_; pH 7.4) to an approximate OD_600_ of 0.6. 3OC8HSL was then added into the suspensions to a final concentration of 1 µM and reacted at 28°C for 2 h. The supernatants from the cultures were also isolated and reacted with 3OC8HSL for 2 h. After incubation, these mixtures were spotted onto the bottoms of solidified agar medium plates, which were cut into separated slices. The *A. tumefaciens* strain NT1 (approximate OD_600_ of 0.6) was then spotted at distances progressively further from the loaded samples. After incubation at 28°C for 24 h, the results were analyzed by monitoring the precipitate color of the *A. tumefaciens* strain NT1. β-galactosidase expression was induced by AHL, which digested X-gal in the medium to give blue colonies. Therefore, the residual AHL in the samples was determined by the distance of the blue colonies on the agar plate.

To further analyze the characteristic of AHL-degrading strains, the supernatant from bacterial cultures was incubated at 60°C for 30 min, and then reacted with 1 µM 3OC8HSL at 28°C for 2 h. The reaction was stopped and residual 3OC8HSL was detected with biosensor.

### Cloning and sequencing of an AHL-degrading gene from *P. byunsanensis* strain 1A01261

A common protocol for genetic manipulation was exploited to construct the genomic library. The DNA fragments, partially digested by *Sau*3A I from the total genomic DNA of strain 1A01261, were ligated into the pUC118 cloning vector (TaKaRa), which was digested by *Bam*H I and dephosphorylated. The genomic library was transformed into *E. coli* DH5α. Subsequently, transformants were grown overnight on LB agar plates containing ampicillin. The colonies were inoculated into fresh LB medium and incubated at 37°C overnight. The colony cultures were then reacted with 250 nM 3OC8HSL for 4 h. The reactions were halted by heating, and residual 3OC8HSL was detected with *A. tumefaciens* NT1. A total of 6,000 clones were screened, and several different transformants manifested AHL-degrading activity. The *E. coli* DH5α harboring positive plasmids were rescreened by inoculation with 2 µM 3OC8HSL. Finally, one positive plasmid, pUC118-*sm20*, was obtained and sequenced. The positive DNA fragment was analyzed with DNASTAR software (DNASTAR, Inc.) and by BLAST (http://www.ncbi.nlm.nih.gov/), BPROM (http://linux1.softberry.com/berry.phtml) and SIGNALP (http://www.expasy.org).

Given that the cloned positive gene encoded a two-domain protein, including the N-terminal GDSL hydrolase domain and C-terminal RND-type transporter domain, we further explored which domain was responsible for the AHL-degrading activity. Thus, we cloned a DNA fragment (named *qsdH*, the abbreviation of quorum sensing degrading hydrolase) encoding the GDSL hydrolase and the open reading frame (ORF) gene, *orf*, into the pUC118 vector. The *orf* gene was amplified from the 1A01261 genome with the following primers: 5′-CCGGAATTC ATGCGCCGACGTCGCCGCGC-3′ and 5′-CGCGGATCCTTAGGATCCCTTTACTATGC-3′ (*Eco*R I and *Bam*H I sites are underlined). The *orf'* gene fragment was amplified with the following primers to process the N-terminal signal sequence to yield the mature ORF protein: 5′-CCGGAATTCGCGCCCCCTTCGCCGCAGTA-3′ and 5′-CGCGGATCCTTAGGATCCCTTTACTATGC-3′. The *qsdH* gene was cloned with the following primers: 5′-CCGGAATTCGCGCCCCCTTCGCCGCAGTA-3′ and 5′-CGCGGATCCTTAGAGGTTTTTCTGCACTG-3′. Finally, these DNA fragments were cloned into pUC118 and transformed into *E. coli* DH5α. The AHL-degrading activity of these transformants was then detected by the biosensor *A. tumefaciens* strain NT1.

### Expression and purification of QsdH protein

As the *qsdH* GDSL hydrolase gene exhibits AHL activity, the gene fragment was amplified without the N-terminal signal sequence using the following primers: 5′-CCGGAATTCGCGCCCCCTTCGCCGCAGTA-3′ and 5′-CCGCTCGAGTTAGAGGTTTTTCTGCACTG-3′ (*Eco*R I and *Xho* I sites are underlined). The PCR products were inserted into the *Eco*R I/*Xho* I site of the pGEX-6p-1 vector and transformed into the *E. coli* BL21 (DE3) expression host.

The *E. coli* BL21 (DE3) strain harboring the recombined plasmid was inoculated into fresh LB medium at 37°C with gentle shaking. After the OD_600_ of the culture reached 0.6, the QsdH-GST fusion protein was induced by the addition of isopropyl-D-thiogalactopyranoside (IPTG) to a final concentration of 0.1 mM followed by further incubation at 22°C for 6 h. The cells were then harvested by centrifugation, washed with PBS buffer, resuspended in PBS buffer, lysed under high pressure and finally centrifuged at 4,000×*g* for 30 min at 4°C to obtain the supernatant. The QsdH-GST protein was bound to glutathione affinity resin with the GST fusion protein purification kit (GE Healthcare). Then, 10 µl of the 3C protease stock solution (10 U/l; GE) diluted with PBS buffer was added into the purification column to digest the GST tag linker overnight after the unbound bacterial proteins completely washed off the column. Finally, the purified protein was stored with an equivalent amount of pure glycerol at −80°C and analyzed with 12% SDS-PAGE.

### Construction of the *qsdH* mutant

The mutants were constructed by mutating specific amino acids according to the protocol of the TransGen Easy Mutagenesis System. Site-directed mutagenesis was carried out with the pGEX-6p*-qsdH* plasmid and pairs of complementary oligonucleotides containing the desired mutation (Table 2). The PCR products (10 µl) were digested with 1 µl of *Dpn* I (TaKaRa) for 2 h at 37°C, which removed the wild-type templates. The digested PCR samples were then transformed into DMT competent cells for further digestion of the parental plasmid. Subsequently, the transformants were screened with ampicillin. Finally, all mutations were verified by double-strand DNA sequencing. Plasmids harboring the desired mutation were transformed into *E. coli* BL21 (DE3) for further protein expression and purification.

**Table 2:** Primers for site-directed mutagenesis.

Primer^1^	Amino acid Substitution	Sequence^2^
S42V-F	Ser42Val	GCTCATGGGGGAC**GTT**TACTCCGCGGGCAA
S42V-R		**AAC** GTCCCCCATGAGCACGTACTGCGGCGA
G83V-F	Gly83Val	TGTCGCGTGCAGC**GTT**GGTGTCGTCGCCGA
G83V-R		**AAC** GCTGCACGCGACATTGGTCAGCTCGGC
N183V-F	Asn183Val	GACCATCGGTGGC**GTT**GACATCGGCTTCAG
N183V-R		**AAC** GCCACCGATGGTCAGGAAGACCGCGTC
N183S-F	Asn183Ser	GACCATCGGTGGC**AGC**GACATCGGCTTCAG
N183S-R		**GCT** GCCACCGATGGTCAGGAAGACCGCGTC

1 F, forward primer; R, reverse primer.

2 The bases changed are shown in boldface type in each primer.

### Analysis of the AHL-degrading activity of QsdH

For *in vivo* assays, *E. coli* DH5α containing constructs of interest were inoculated into LB medium containing 100 µg/ml ampicillin and grown overnight followed by incubation with 3OC8HSL to a final concentration of 2 µM for 4 h at 37°C. Subsequently, the mixtures were heated in a 95°C water bath and detected by *A. tumefaciens* strain NT1. For the purified protein assay, a standard reaction mixture containing 90 µl of 200 mM NaH_2_PO_4_-Na_2_HPO_4_ buffer (pH 7.3), 5 µl of 100 µM 3OC8HSL and 5 µl of the purified protein (2.5 µg) was used. A mixture composed of 95 µl of the same buffer and 5 µl of 100 µM 3OC8HSL was used as a control. After incubation at 40°C for 30 min, the mixture was heated in boiling water for 5 min and detected with *A. tumefaciens* strain NT1.

### Enzyme assay

HPLC was performed according to a previously described method [30]. The amount of AHL was estimated by comparing the reduction in peak areas with an AHL solution of known concentration and was further calibrated with pure AHLs in the concentration range of 100 nM to 2, 000 nM. The optimal temperature of activiy was measured at 20°C to 60°C, under enzymatic reaction system of 5 µl of purified QsdH in 490 µl of NaH_2_PO_4_- Na_2_HPO_4_ buffer (pH 7.3) and 5 µl of 100 mM 3OC8HSL; the non-enzymatic degradation of 3OC8HSL in reaction system at different temperatures were detected as controls. After incubation of 30 min, the reactions were stopped, and the AHLs were extracted three times with an equal volume of ethyl acetate. The combined organic phase in sample was evaporated with nitrogen to dryness, and then the residual 3OC8HSL were detected by HPLC. For HPLC analysis, the samples were dissolved in 500 µl of column buffer (methanol: water at 4∶6; v/v), which was introduced into a symmetry C_18_ reverse-phase column (4.6 mm×250 mm) (Kromasil C18-5u) and detected with an UV/visible light (Waters) detector set at 200 nm. Fractions were eluted with a mobile phase of methanol: water (60∶40; v/v) at a flow rate of 1 ml/min. To detect the substrate specificity of purified QsdH for AHL-degrading activity, 5 µl of purified QsdH reacted with different AHLs (C4HSL, C6HSL, C8HSL, C10HSL, C12HSL, C14HSL, 3OC6HSL, and 3OC8HSL) respectively. After incubation for 30 min at 40°C, the reaction was halted with a 95°C water bath. AHLs were extracted three times with an equal volume of ethyl acetate, and the combined organic phase was then evaporated. AHLs (1 mM) were incubated in the same enzyme reaction buffer at 40°C for 30 min and extracted as a control. Samples were dissolved in 500 µl of column buffer and detected by HPLC. To investigate the effects of divalent ions on enzymatic activity, several divalent ions at final concentrations of 0.2 and 10 mM were added into the enzymatic reaction system. After incubating 1 mM 3OC8HSL (final concentration) with 5 µl of purified QsdH in a reaction buffer containing different divalent ions for 30 min at 40°C, the residual 3OC8HSL were extracted three times with ethyl acetate respectively. The combined organic phase was then evaporated to dryness. The samples were redissolved in methanol, and 3OC8HSL was detected and quantified by HPLC.

### Determining the mechanism of AHL degradation of QsdH

To determine the chemical structure of products of the AHLs and QsdH, 3OC8HSL was digested by QsdH, and the reaction products were analyzed using HPLC and ESI-MS. A 5 µl of the purified QsdH (2.5 µg) enzyme solution with 1 mM 3OC8HSL (final concentration) was added to the Na_2_HPO_4_-KH_2_PO_4_ reaction buffer (pH 7.3). After incubation for 30 min at 40°C, the reaction was halted with a 95°C water bath, and the reacted products were extracted three times with ethyl acetate. The combined organic phase was then evaporated to dryness. The samples were dissolved in methanol and introduced into a symmetry C_18_ reverse-phase column for HPLC analysis. The lactonolysis product of 3OC8HSL, 3-oxooctanoyl-L-homoserine, was prepared by hydrolysis of the lactone ring in alkaline buffer as previously described [30]. 3OC8HSL (1 mM) was digested in a solution containing 200 µl of dimethyl sulphoxide (DMSO) and 300 µl of 1 M NaOH for 6 h at 37°C, and the mixture was then adjusted with H_3_PO_4_ to a pH of 5.0. Next, 3-oxooctanoyl-L-homoserine was extracted three times with ethyl acetate and evaporated to dryness. The sample was redissolved in a methanol: water solution and purified by HPLC using a C_18_ reverse-phase column. ESI-MS was performed with an API5000 triple-quadrupole instrument from Applied Biosystems (USA). Samples were dissolved in methanol and ionized by negative-ion electrospray.

### The production of AHLs and the expression of *qsdH* in *P. byunsanensis* strain 1A01261

To determine whether the production of AHLs was reliable to the cell density of *P. byunsanensis* strain 1A01261, we extracted AHLs from culture of strain 1A01261. The amount of AHLs was detected by HPLC with standard AHLs as controls. Strain 1A01261 was cultured in 2 L modified LB media (LB medium with 3% (w/v) NaCl) at 25°C. At 24-h intervals a 50-ml aliquot of the culture was centrifuged, and the supernatant was extracted 3 times with 50 ml of chloroform. The extract was evaporated, dried, and dissolved in 1 ml of methanol for HPLC analysis. The growth of the bacteria was calculated by measuring the absorbance at 600 nm; bacteria was inoculated into medium and cultured for 0 h as a control.

To detect the expression of QsdH in strain 1A01261, we identified the expression of *qsdH* in RNA level. At 24-h intervals 2 ml culture of bacteria incubated in modified LB medium was centrifuged with a control of uncultured bacteria, and RNA in cell was extracted by Trizol reagent as the method on Operating instruction in kit (Invitrogen, USA). After removal of DNA template in samples, cDNA was amplified from RNA with random 9 mers by RT-PCR as the method annexed in RT-PCR kit (Ferments, USA). Finally, the gene *qsdH* was amplified with specific primers of 5′-GCGCCCCCTTCGCCGCAGTA-3′ and 5′-TTAGAGGTTTTTCTGCACTG-3′.

### Virulence tests

*E. carotovora* as a plant pathogen produces and secretes exoenzymes that act as virulence determinants for soft rot diseases of various plants. To test the effect of AHL-lactonase on bacterial infection, we detect the prevention of bacterial infection of *E. coli* BL21 containing over-producing QsdH. After *E. carotovora* strain SCG1 was cultivated until the OD_600_ was 1.0, 200 µl of culture was centrifuged and then diluted with saline solution (0.15 M NaCl) to a final OD_600_ of 0.1. Recombinant *E. coli* carrying pGEX-6p-*qsdH* and *E. coli* carrying pGEX-6p-1 were harvested 6 h after IPTG induction at 22°C, and resuspended in saline solution to a final OD_600_ of 3.0. After bacteria were diluted, equal volumes of *E. carotovora* SCG1 and recombinant *E. coli* were mixed respectively. Equal volumes of 0.15 M NaCl solution incubated with *E. carotovora* SCG1 as a negative control. 20 µl of the mixtures were loaded onto potato slices and incubated at 30°C for 24 h. In addition, 20 µl of *E. coli* contaning pGEX-6p-*qsdH* or pGEX-6p-1, and NaCl were loaded onto potato slices respectively as positive controls. Watery rotten lesions around inoculation sites were observed as evidence of the activation of virulence, the area of rotten lesions in potato representing amount of virulence.

### Nucleotide sequence accession number

The nucleotide sequences of *orf* gene, encoding the RND-type efflux transporter protein from *P. byunsanensis* strain 1A01261 have been deposited in the DDBJ/EMBL/GenBank databases under accession number JX392407.

## Results

### Identification of AHL-degrading activity of marine bacteria *P. byunsanensis* strain 1A01261

One thousand independent isolates from the Marine Culture Collection of China (MCCC) were incubated with 3OC8HSL, and the AHL-degrading activities were detected on agar plates with *A. tumefaciens* NT1. Among these isolates, ten strains displayed different AHL-degrading activity, including *P. byunsanensis*, *Alcanivorax dieselolei*, *Bacillus cereus*, *Alcanivorax venusti* and *Marinobacter hydrocarbonoclasticus*, *Halomonas sp.* and so on. In which *P. byunsanensis* (MCCC1A01261) displayed high AHL-degrading activity. Moreover, the main AHL-degrading activity of strain 1A01261 was exhibited in supernatant of the marine strain. In addition, the AHL-degrading activity of *P. byunsanensis* strain 1A01261 was thermostable because it still harbored high activity after the supernatant of bacterial culture incubated at 60°C for 30 min. Finally, *P. byunsanensis* spp. represents a novel species within the *Pseudoalteromonas* genus and was first isolated in 2009 [31], study about which was little. Therefore, as a novel material, *P. byunsanensis* had the potential value for study.

### Cloning and characterization of the AHL-degrading gene from *P. byunsanensis* strain 1A01261

**Figure 1:**
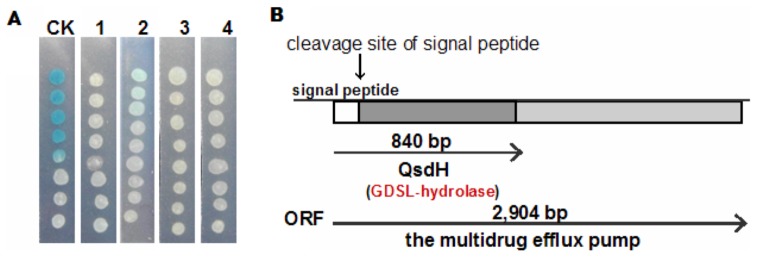
Screening an AHL-degrading activity gene *qsdH* and the physical map of *qsdH* locus. (A) The AHL-degrading activity of *E. coli* DH5α harboring different plasmids detected with biosensor *A. tumefaciens* strain NT1. CK, pUC118, used as a control; 1, pUC118-*sm20*, which was the screened positive transformant; 2, pUC118-*orf* (encodes the full ORF protein); 3, pUC118-*orf'* (encodes the ORF protein removing the N-terminal signal peptide); and 4, pUC118-*qsdH*. (B) A schematic representation of the complete ORF, which contains an N-terminal GDSL hydrolase domain and a RND-type multidrug efflux protein domain with the GDSL-lipolytic enzyme named QsdH.

After screening approximately 6, 000 transformants from a genomic library of *P. byunsanensis* 1A01261, several positive clones were obtained and then rescreened with the biosensor *A. tumefaciens* NT1. The positive transformant-containing plasmid, pUC118-*sm20*, displayed AHL- degrading activity with complete inactivation of 2 µM 3OC8HSL in 4 h (Fig. 1A). Sequencing analysis demonstrated that pUC118-*sm20* contained a cloned genomic fragment of 3, 116 bp, which encompassed one complete open reading frame (ORF) (Fig. 1B). The *orf* gene was predicted to encode a protein of 968 amino acids with a predicted isoelectric point at 4.84. BLAST analysis suggested that this gene encompassed the following two catalytic domains: an N-terminal GDSL hydrolase fold family domain (the highly conserved residues ‘Gly-Asp-Ser-X’ around the catalytic site) and a C-terminal RND-type multidrug efflux transporter domain.

**Figure 2:**
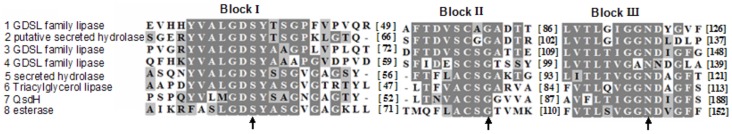
Multiple alignment of the deduced amino acid sequence of *qsdH* and GDSL-like lipase/esterase. 1, GDSL family lipase from *Amycolatopsis mediterranei* U32 (GI: 300788557); 2, a putative secreted hydrolases from *Streptomyces sp.* Tu6071 (GI: 333028773); 3, a GDSL family lipase from *Pseudonocardia sp.* P1 (GI: 324999532); 4, a GDSL family lipase from *Segniliparus rotundus* DSM 44985(GI: 296394487); 5, a secreted hydrolases from *Streptomyces sp.* AA4 (GI: 302529957); 6, a triacylglycerol lipase from *Kribbella flavida* DSM 17836 (GI: 284031490) and 8, a esterase from *Coccidioides posadasii* str. Silveira (GI: 320039410). Conserved residues are shaded in gray, and the catalytic amino acid residues (Ser, Gly and Asn) in the consensus sequences are marked.

The measurement of the AHL-degrading activities of *E. coli* DH5α harboring different recombinant plasmids, including pUC118-*orf*, pUC118-*orf'* and pUC118-*qsdH*, elucidated that all three of the transformants completely digested 2 µM 3OC8HSL within 4 h (Fig. 1A). This result suggested that the catalytic region containing a GDSL hydrolase possessed similar enzymatic activity to the full-length ORF. Among enzymes with demonstrated functions, QsdH was most similar to the secreted hydrolase from *Streptomyces* spp. AA4 (GI 302529957), sharing 47% identity at the amino acid level. QsdH also shared 45% identity with the triacylglycerol lipase from *Kribbella flavida* DSM17836 (GI 284031490) and 34% identity with EstA from *Pseudoalteromonas* spp. 643A (GI 194369063). However, the tertiary fold of the GDSL hydrolase family is substantially different from that of the alpha/beta hydrolase family, which is unique among all known hydrolases. The active site of GDSL hydrolase proteins contains two of the three components of a typical Ser-His-Asp (Glu) triad found in other serine hydrolases but may lack the carboxylic acid [32], [33]. QsdH was found to contain three conserved residues in I, II and III blocks, which formed the oxyanion holes (Ser42-Gly83-Asn183) of the GDSL hydrolase fold according to the analysis of a multiple protein sequence alignment (Fig. 2). However, QsdH was found to lack the catalytic His and Asp in block V.

### Enzymatic characterization of the AHL-degrading activity of QsdH

**Figure 3:**
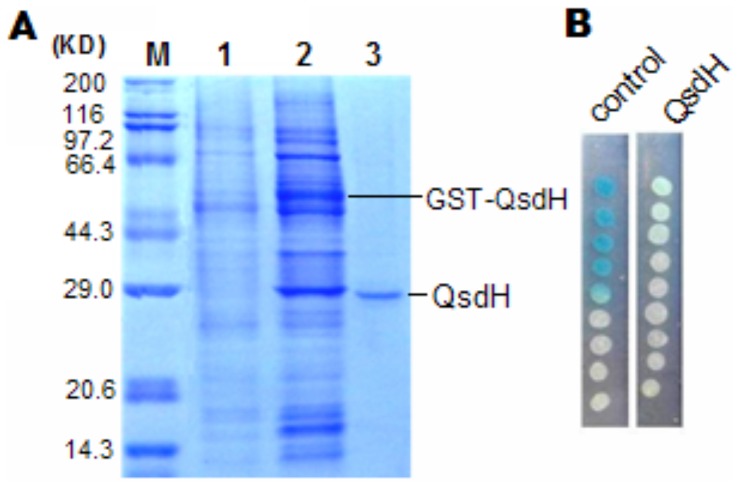
Purification of QsdH and identification of AHL-degrading activity of QsdH. (A) Analysis of the expression and purification of QsdH protein by 12% SDS-PAGE. M is the standard molecular weight markers (TaKaRa); Lanes 1 and 2 are uninduced and induced cell lysates of *E. coli* BL21 (DE3) harboring pGEX-6p-*qsdH*, respectively. Lane 3 is the purified QsdH from *P. byunsanensis* 1A01261. The protein bands of GST-QsdH and QsdH are marked respectively. (B) AHL-degrading activity of purified QsdH. The solution of purified QsdH was mixed into the reaction buffer containing 5 µM 3OC8HSL (final concentration) and incubated at 40°C for 30 min. The residual 3OC8HSL was detected by *A. tumefaciens* strain NT1. The control consisted of 5 µM 3OC8HSL in reaction buffer incubated at 40°C for 30 min.

**Figure 4:**
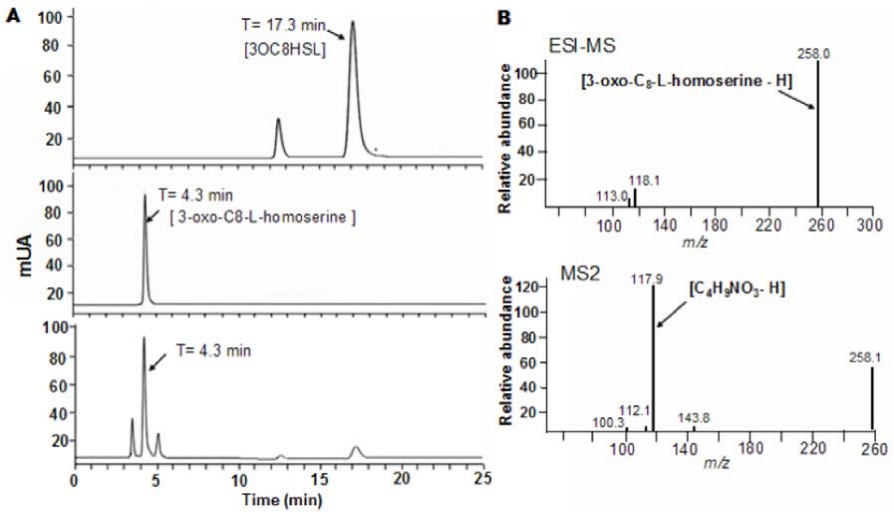
HPLC and ESI-MS spectrometry analysis of the QsdH-catalyzed OHHL product. (A) HPLC analysis of the AHL lactonase digestion product of 3OC8HSL. (Upper) HPLC profile of 3OC8HSL (retention time of 17.3 min). The minor peak at 12.7 min was the sodium salt of 3OC8HSL based on MS analysis (data not shown). (Middle) HPLC profile of 3OC8HSL hydrolyzed by NaOH, which released the ring-opened product of 3OC8HSL with a retention time of 4.3 min. (Lower) HPLC profile showed that 3OC8HSL digestion by AHL lactonase resulted in products with a retention time of 4.3 min. (B) ESI-MS and MS2 analysis of the hydrolysis product of 3OC8HSL by QsdH. (Upper) ESI-MS analysis of the 4.3 min HPLC fragment of enzymatic-digested products showed a quasimolecular (M–H) ion substrate at *m/z* of 258.1. (Lower) MS2 analysis of the parent ion at *m/z* of 258 by tandem mass spectrometry showed a daughter ion of 118.1.

Because the *qsdH* gene lacking the signal peptide still manifested AHL-degrading activity, the mature QsdH was purified using GST affinity chromatography. The calculated molecular weight of 27.0 kDa was consistent with the molecular mass determined by 12% SDS-PAGE (Fig. 3A). An examination of the AHL-degrading activity showed that purified QsdH was able to completely degrade 5 µM 3OC8HSL at 40°C for 30 min (Fig. 3B). To determine if QsdH degraded AHL by acting as a lactonase or as an acylase, 3OC8HSL was digested with purified QsdH, and the reaction products were then analyzed via HPLC and ESI-MS. Fractionation of pure 3OC8HSL revealed one major HPLC peak with a retention time of 17.3 min and a minor peak with a retention time of 12.7 min, which was the sodium salt of 3OC8HSL based on MS analysis (data not shown). Meanwhile, the ring-opened product of 3OC8HSL, which was prepared by hydrolyzing 3OC8HSL with 1 M NaOH, displayed a HPLC peak with a retention time of approximately 4.3 min (Fig. 4A). The enzyme digestion products contained a similar peak with a retention time of 4.3 min and a peak of undigested 3OC8HSL with a retention time of 17.3 min. To confirm product identity, enzyme-digested product fragments were collected for ESI-MS analysis. The results demonstrated that the 4.3 min HPLC fragment was a substrate of a quasimolecular (M-H) ion at *m/z* of 258.1 (Fig. 4B), which indicated that an enzymatic digestion of 3OC8HSL led to a mass increase of 18 in its products. In addition, MS2 analysis of the parent ion at *m/z* of 258 by tandem mass spectrometry showed a daughter ion of 118.1 (Fig. 4B; down column), which was consistent with the formula of C_4_H_9_NO_3_ (homoserine; M-H ion m/z of 118.1). Together, these data demonstrated that *qsdH* encodes an AHL lactonase that hydrolyzes the homoserine lactone ring of 3OC8HSL, thus releasing *N*-acyl-homoserine.

**Figure 5:**
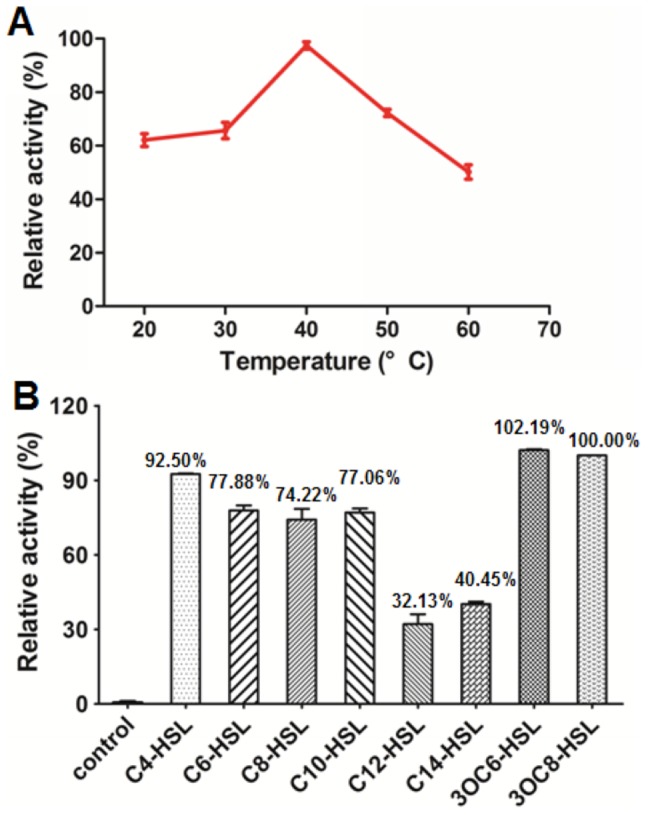
Enzymatic characteristics of QsdH for AHL-degrading activity. (A) Effect of temperature on enzyme activity. The activity of QsdH to 3OC8HSL was measured at temperatures ranging from 20°C to 60°C. The residual 3OC8HSL in reaction system without enzyme at different temperatures was detected as controls. The highest activity at 40°C was defined as 100%. (B) Detect the substrate specificity of AHL-lactonase QsdH. 1 mM different substrate incubated with purified QsdH, and then the residual substrate was quantified by HPLC. Data are the means of 3 measurements. The activity of QsdH toward 3OC8HSL was defined as 100%. The 3OC8HSL-degrading activity without QsdH is represented as the control.

QsdH exhibited activity at temperature ranging from 20°C to 60°C, reaching its optimum activity at 40°C after incubation of 30 min at pH 7.3 using 3OC8HSL as a substrate (Fig. 5A). The substrate specificity of QsdH was determined by detecting the enzymatic activity against a range of AHLs with or without substitution of carbon 3 by HPLC. Figure 5B shows that QsdH exhibited high relative activities to all tested AHLs but worked better with short- and medium-chain acyl homoserine lactones (such as C4HSL, C6HSL, C8HSL and C10HSL) than long-chain acyl homoserine lactones (C12HSL and C14HSL). A 3-oxo substitution of AHLs (3OC8HSL and 3OC6HSL) was detected and efficiently degraded by QsdH.

To determine the effect of divalent ions on the AHL-degrading activity of QsdH, several divalent ions (Zn^2+^, Cu^2+^, Ca^2+^, Mg^2+^, Ni^2+^, Ba^2+^, Sr^2+^ and Mn^2+^) were added into the enzyme reaction system, and the AHL-inactivating function of QsdH was evaluated by HPLC. The effects of two concentrations (0.2 and 10 mM) of various metal ions on the enzymatic activity of QsdH are shown in Table 3. The presence of all assayed metal ions at 0.2 mM showed a slight enhancement of the AHL-degrading activity of QsdH, which was approximately 5–15% greater than the control. However, most of the tested metal ions at 10 mM inhibited the AHL-degrading activity of QsdH.

**Table 3:** Effects of metal ions on QsdH activity for 3OC8HSL.

Metal ion	Relative activity (%)	
	10 mM	0.2 mM
None	100	100
Zn^2+^	64.57	106.46
Ni^2+^	74.63	109.02
Cu^2+^	75.12	105.55
Ba^2+^	95.85	108.05
Mg^2+^	90.88	107.58
Sr^2+^	84.43	107.08
Ca^2+^	103.63	113.44
Mn^2+^	87.97	105.42

### QsdH is a member of the AHL lactonase family

Sequence alignment was performed with QsdH and previously studied AHL lactonases. QsdH shared a low similarity to known lactonases. For example, the sequence similarity of QsdH to several AHL lactonases was as follows: 26% similarity to AiiA (*Bacillus* spp.), 24% similarity to QsdA (*R. erythropolis* W2), 25% similarity to AhlD (*Arthrobacter* spp.), 32% similarity to AiiM (*M. testaceum*), and no similarity to AidH (*Oclrobacterum* spp.), AiiB and AttM (*A. tumefaciens*). The main zinc-binding motif of the conserved HXHXDH sequence is found in many AHL lactonases. For example, AidH and AiiM have a α/β hydrolase fold in which a conserved G-X-Nuc-X-G or histidine is critical to the AHL-degrading activity. However, these conserved motifs were not found in QsdH, which suggested that QsdH was quite different from the abovementioned AHL lactonases. EstA, a homologous gene of QsdH from *Pseudoalteromonas* spp. 643A, has been shown to have activity for esters of short- to medium-chain (C_4_ and C_10_) fatty acids and to not have activity for long-chain fatty acid esters or any lactones [34]. Meanwhile, the EstA of *Serratia liquefaciens* MG1, which is a GDSL-lipolytic enzyme, has been shown to hydrolyze only short-chain naphthol esters with a maximum of six carbons [35]. Together, these data suggest that the functional QsdH may not be present in the two strains.

### The conserved serine, glycine and asparagine residues are required for QsdH activity

**Figure 6:**
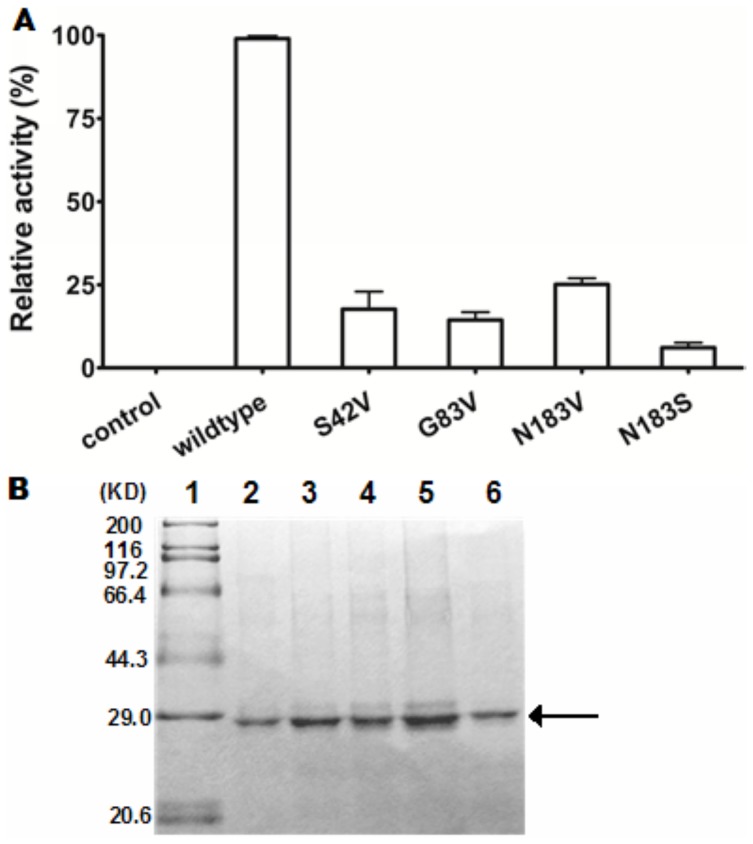
Conserved residues are important for the enzymatic activity of QsdH. (A) Site-directed mutagenesis replacing Ser42, Gly83, and Asn183 with Val and replacing Asn183 with Ser reduced QsdH activity. The purified mutants were incubated with 1 mM 3OC8HSL, and the residual 3OC8HSL was detected by HPLC. Data are the mean values of three measurements. The activity of wild-type QsdH toward 3OC8HSL was defined as 100%. (B) Substitution mutants of QsdH made stable proteins. The mutants were expressed and purified in the same manner as wild-type QsdH, and purified mutants were analyzed by SDS-PAGE. 1, standard molecular weight markers (TaKaRa); 2–6, purified proteins of wild-type QsdH and mutants S42V, Gly83V, N183V and N183S, respectively.

Site-directed mutagenesis was used for exploring the roles of these conserved residues (Ser42, Gly83 and Asn183) in three conserved blocks. We replaced Ser42, Gly83 and Asn183 with a Val residue and replaced Asn183 with a Ser residue. These mutants were then purified in a manner indistinguishable from that of wild-type QsdH. The activities for AHL inactivation of these mutants are shown in Figure 6A. The enzymatic activity of the S42V, G83V, N183V and N183S mutants was drastically reduced. These results suggested that the Ser, Gly and Asn oxyanion residues in the conserved blocks have important roles in QsdH activity and that the loss of activity is due to changes in the biochemical properties of the mutant proteins but not in the expression conditions (Fig. 6B).

### In silico prediction of QsdH localization within the cell

**Figure 7:**
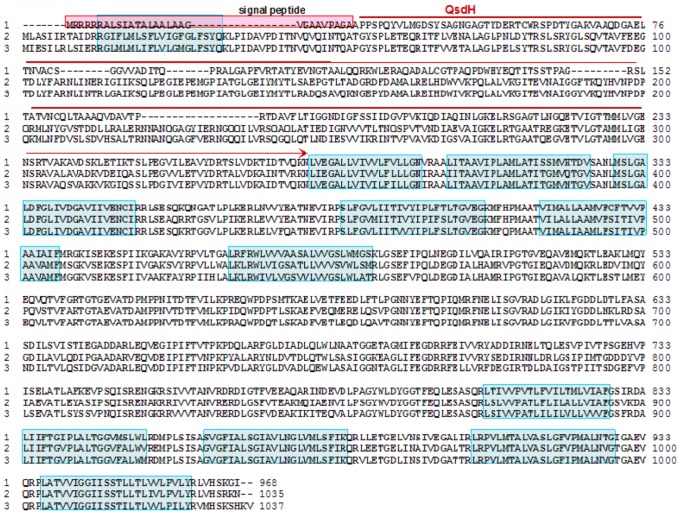
QsdH is localized in the inner membrane of a RND-type multidrug efflux transporter. The alignment of the ORF protein with two multidrug efflux pumps was showed. 1, ORF protein; 2, CzcA family heavy metal efflux protein from *Alteromonas* spp. SN2 (GI: 333892934); and 3, CzcA family heavy metal efflux protein from *Pseudoalteromonas atlantica* T6c (GI: 109898348). Structural predictions illustrate 12 transmembrane helices with two perisplasmic loops located between TM1 and TM2 and between TM7 and TM8 in the ORF protein (RND-type efflux transporter). The 12 transmembrane helices are shaded with a green box, and the typical signal peptide is marked in the ORF protein with a red box. Using *in silico* prediction, the GDSL hyrolase, QsdH, was predicted to form the first perisplasmic loop of the RND-type multidrug efflux transporter.

A BLAST search revealed that the amino acid sequences deduced for the ORF protein showed a high identity with the CzcA family metal efflux proteins from *Alteromonas* spp. SN2 and *Glaciecola* spp. HTCC2999, as well as the RND divalent metal cation efflux transporters from *Collimonas fungivorans* Ter331 and *Alcanivorax borkumensis* SK2 (Fig. 7). These results demonstrated that the ORF protein belongs to a RND-type transporter protein family, which is one part of a three-component multidrug efflux pump. Subsequent determination of the cellular location of the RND-type transporter protein by the SPORT computer program revealed that this protein is located at the inner membrane. These data suggested that the N-terminal GDSL hydrolase, QsdH, is located on the cytoplasmic membrane of the multidrug efflux protein. As predicted by the SIGNALP program, SOPM program and secondary structure prediction method (http://pbil.ibcp.fr/htm/index.php), the N-terminal 29 amino acids of QsdH are a signal sequence. The secondary structure of the signal peptide domain was found to have the following features: 1) N-domain with positively charged amino acids (MRRR); 2) hydrophobic region (H-domain) of neutral amino acids (RRALSIATALAALAAGVG); and 3) signal peptidase recognition sites (AGA) with cleavage occurring after the second Ala. This type of signal peptide is common in previously studied autotransporter proteins in gram-negative bacteria [36]. In addition, domain analysis via SMART (http://smart.embl-heidelberg.de/) predicted that the full length ORF protein has 12 transmembrane domains (α-helix) leaving both the amino and carboxyl termini on the cytoplasmic side of the inner membrane, and that two periplasmic loops of a long hydrophilic domain containing 300 amino acids are formed between TM1 (transmembrane 1) and TM2 and between TM7 and TM8. According to sequence alignment among RND-type transporter proteins, the GDSL hydrolase, QsdH, is located in the first periplasmic loop. Based on these predictions and analyses, we concluded that QsdH may be inserted into a RND-type inner membrane, which is exposed to the exterior periplasm.

### The production of AHLs and the expression of *qsdH* in *P. byunsanensis* strain 1A01261

**Figure 8:**
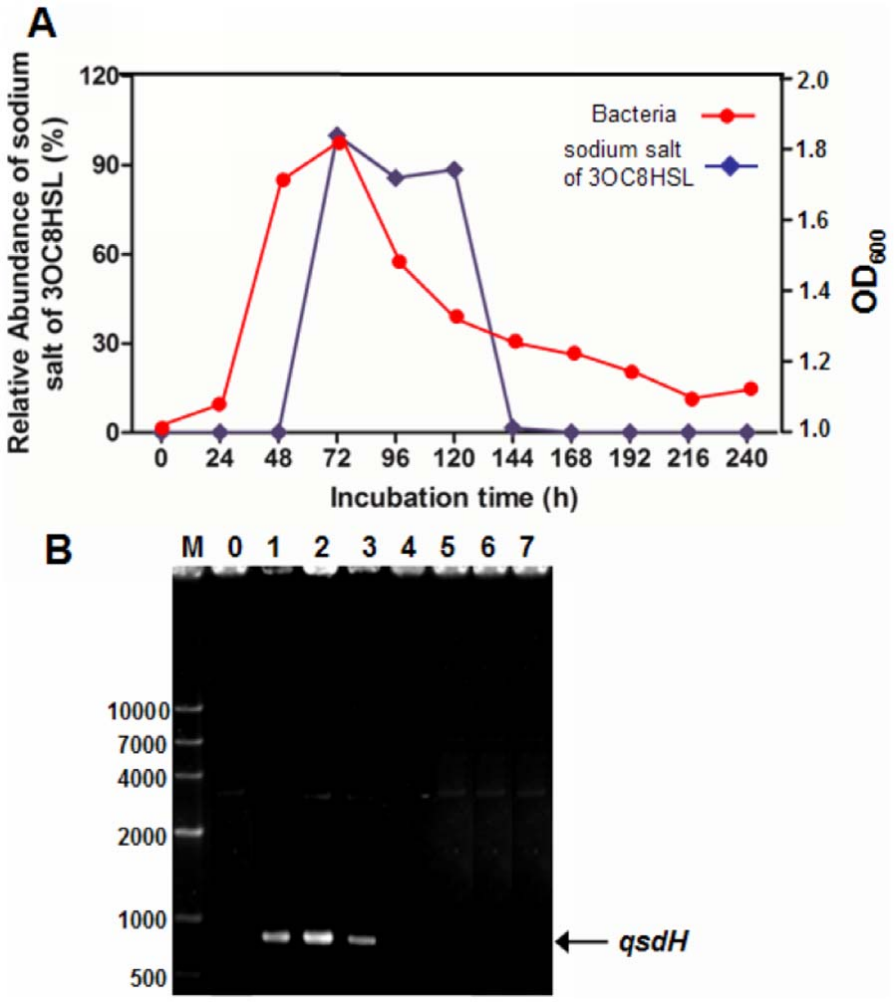
Production of OOHL and QsdH expression in *P. byunsanensis.* (A) Time Courses for the Growth of *P. byunsanensis* Strain 1A01261, and the Production of N-Acylhomoserine Lactones (AHLs). The growth of the bacteria was calculated by measuring the absorbance at 600 nm. Strain 1A01261 was cultured in modified LB media (LB medium with 3% (w/v) NaCl) at 25°C. At 24-h intervals a 50-ml aliquot of the culture was centrifuged, and AHLs were extracted from supernatants of cultures, which were measured by HPLC. The extracted AHL was identified as sodium salt of 3OC8HSL compared with control. Maximum abundance of sodium salt of 3OC8HSL was measured at 72 h, which was defined as 100%. (B) The *qsdH* expression was detected with RT-PCR. M, DNA marker (TaKaRa); 0–7, *qsdH* amplified from total RNA from cultures of *P. byunsanensis* strain 1A01261, with strain incubating for 7 days; the culture for 24-h intervals as a sample, the PCR products amplified from samples was detected by agar gel electrophoresis.

The time course for the growth of bacteria and production of AHLs was shown in Fig. 8A. In this study, bacterial growth of *P. byunsanensis* strain 1A01261 reached the high point at 72 h, and decreased thereafter. At 24-h intervals 50 ml culture of strain was centrifuged and detected the production of AHL by HPLC. The result demonstrated that the production of 3OC8HSL (sodium salt of 3OC8HSL was detected in bacterial culture of modified LB media which contain high concentration of NaCl) was increased at 48 h, and decreased thereafter till to disappear at 144 h.

We also detected the *qsdH* expression at 24-intervel culture of strain 1A01261. The result demonstrated that *qsdH* expression was appeared at 24 h to 72 h, on which the cell grew in log phrase, (Fig. 8B). After 72 h, the *qsdH* expression was not detected, and now the signal molecule, 3OC8HSL, was accumulated.

### QsdH-overproducing *E. coli* for the attenuation of plant-pathogenic *E. carotovora*

**Figure 9:**
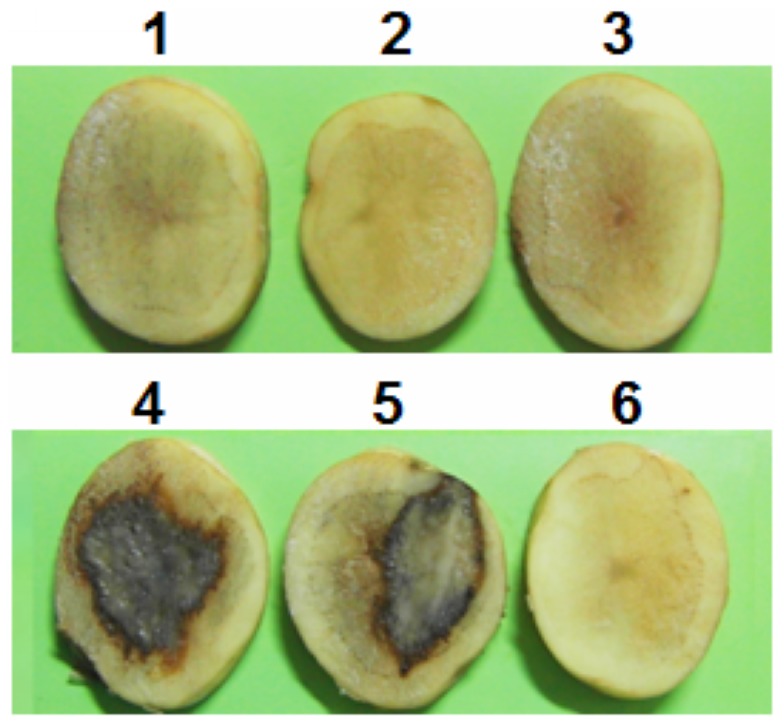
Attenuation of potato pathogenicity of *Erwinia carotovora* by recombinant QsdH- producing *E. coli*. 1, saline solution; 2, *E. coli* carrying pGEX-6p-1; 3, *E. coli* carrying pGEX-6p-*qsdH*; 4, *E. carotovora*; 5, mixture of *E. carotovora* and *E. coli* carrying pGEX-6p-1; 6, mixture of *E. carotovora* and *E. coli* carrying pGEX-6p-*qsdH*.

The QsdH-overproducing *E. coli* mixed with *E. carotovora* SCG1, and displayed a decrease of watery rotten lesions in the potato slices. In contrast, attenuation was not observed in recombinant *E. coli* containing plasmid pGEX-6p-1 (Figure 9). This result showed that the potato virulence of *E. carotovora* whose virulence was regulated by AHLs was attenuated by AHL-degrading enzyme-overproducing *E. coli*. This suggested that the enzymatic quenching of AHL quorum-sensing signals by QsdH is a feasible approach for prevention of bacterial infection, which might has potential to use for the control of gram-negative plant-pathogenic bacteria in the production of crops.

## Discussion

The *P. byunsanensis* strain 1A01261 produces an enzyme that can inactivate a broad range of AHLs with or without 3-oxo substitutions. In this study, the *orf* gene encoding an AHL inactivation protein was cloned and fully sequenced. Sequence analyses with BLAST suggested that this protein contained the following two distinct domains: an N-terminal catalytic domain that harbored a GDSL hydrolase domain and a C-terminal RND-type efflux pump domain. Moreover, removal of the C-terminal RND-type transporter membrane domain rendered a stand-alone GDSL hydrolase domain, which still retained full AHL-degrading activity. The purified QsdH exhibited broad spectrum substrate specificity to AHLs. The HPLC and ESI-MS analysis demonstrated that QsdH was an AHL lactonase able to hydrolyze the homoserine lactone ring to release *N*-acyl-homoserine. Moreover, QsdH shared both low similarity to known AHL lactonases and low identity to reported GDSL-like hydrolases.

These enzymes of the GDSL hydrolase family have two obvious features as follows: 1) the enzymes share little sequence homology with true lipases; and 2) unlike other lipases where the GXSXG motif is near the center, the serine-containing motif of the GDSL subfamily (Gly-Asp-Ser-X) is closer to the N-terminus [32], [37]. GDSL-like hydrolases are useful to many branches of industry because of their multifunctional properties, such as board substrate specificity, regiospecificity, flexibility of active site and capability of changing the conformation of the substrate after binding [32], [37], [38]. Riedel et al. reported that the GDSL esterase, EstA, which is located proximal to the *swr* quorum-sensing system of *Serratia liquefaciens* MG1, is required for AHL biosynthesis when cells are grown on certain lipidic substrates to provide fatty acids [35]. In the present study, however, the GDSL hydrolase, QsdH, conferred strong enzymatic activity to all tested AHLs, which suggested that *N*-acyl-homoserine lactones are the first reported substrates of the GDSL hydrolase family. In addition, mutagenesis of the conserved catalytic residues (Ser, Gly and Asn) in the three conserved regions (blocks I, II, III) led to greatly reduced lactonase activity of QsdH, which indicated that QsdH was clearly a member of the GDSL hydrolase family. In particular, these conserved active site residues had pivotal roles in the enzymatic activity of AHL inactivation. GDSL hydrolases are not known to require cofactors for their activity [32], which was consistent with our observations that the lactonase activity of QsdH did not rely on any divalent ion.

Intriguingly, the GDSL hydrolase, QsdH, was found to be integrated into the inner membrane protein, RND-type efflux transporter, which is involved in multidrug transportation. Recently, several GDSL family lipolytic enzymes covalently attached to outer membranes and secreted by gram-negative bacteria have been designated to be members of an autotransporter family [35], [39], [40], [41]. To our knowledge, this is the first report of a GDSL hydrolase anchored with a C-terminal cytoplasmic membrane of the multidrug efflux transporter protein in the *Pseudoalteromonas* genus. The following three distinct domains have been suggested to be present in nearly all autotransporter proteins: amino-terminal leader peptide; surface-localized mature protein; and carboxy-terminal domain that mediate secretion through the outer membrane. Moreover, prior investigations have identified the key structural features of the amino-terminal leader peptide of autotransporter proteins involved in exporting precursors through the inner membrane in a Sec-independent manner [36]. In the present study, a typical signal peptide of inner membrane proteins was predicted in the N-terminus of QsdH and was predicted to constitute the first transmembrane structure of the multidrug protein. These data revealed that the signal peptide may lead to the translocation of QsdH to the inner membrane without being cleaved.

The RND-type transporter is one part of the three-component efflux pump, which has a significant role in multidrug efflux. These tripartite pumps are composed of an integral inner membrane drug-proton antiporter of the RND family of exporters, a channel-forming outer membrane efflux protein (or outer membrane factor; OMF) and a periplasmic membrane fusion protein (MFP) [23]. Studies over the past few years have documented that these multiprotein complexes transport a wide variety of substrates, including antibiotics, dyes, detergents and host-derived molecules, from the periplasm to the extracellular space [42]. However, previous investigations have outlined that the efflux of long-chain AHLs used to synchronize quorum sensing in many gram-negative bacteria depends on active transportation mechanisms [24], [25], [26], [27], [28]. Furthermore, previous studies have illustrated that the integral inner membrane RND component of three-component multidrug efflux systems defines the substrate selectivity of these efflux systems [43], [44]. The inner membrane RND-type transporter contains 12 membrane-spanning helices and 2 periplasmic loops of approximately 300 amino acids between helixes 1 and 2 and between helixes 7 and 8 [45]. More recently, the importance of the periplasmic loops for substrate recognition and transport in *P. aeruginosa* has been demonstrated [43], [44], [46]. In this study, the GDSL hydrolase, QsdH, lies in the first periplasmic loop. Thus, the possibility that AHLs are digested by QsdH when these signal molecules influx or efflux through the inner membrane should not be excluded.

The *Pseudoalteromonas* genus is a marine group of bacteria known to influence boil formation in various marine econiches [47]. *P. byunsanensis* spp. represents a novel species within the *Pseudoalteromonas* genus and was first isolated from tidal sediment in Korea. The phenotypic features of *P. byunsanensis* spp. are similar to those of *Pseudoalteromonas phenolica* and *Pseudoalteromonas luteoviolacea* exhibiting alkaline phosphatase, esterase (C_4_), esterase lipase (C_8_) and leucine arylamidase activities [31]. Several phenotypic characterizations of the *Pseudoalteromonas* spp. marine bacterium associated this species with quorum sensing. For example, the antibacterial activity of *Pseudoalteromonas* spp. NJ6-3-1 is regulated by quorum sensing [48]. Additionally, N-(3-oxooctanoyl)-homoserine lactone has been shown to be a signaling molecule involved in the production of violacein of *Pseudoalteromonas* spp. 520P1 [49]. Furthermore, the expression of five enzymes (VioA-VioE) is responsible for synthesizing violacein of *Pseudoalteromonas* spp. 520P1, which is regulated by a quorum-sensing mechanism [50]. In this study, bacterial growth of *P. byunsanensis* strain 1A01261 reached the high point at 72 h, and decreased thereafter. Moreover, the production of 3OC8HSL was increased at 48 h, and decreased thereafter till to disappear at 144 h (Fig. 8A), suggesting that AHL was accumulated after the cell growth on log phrase. Detect *qsdH* expression in strain 1A01261, and the result showed that *qsdH* expression was appeared at 24 h to 72 h, on which the cell grew in log phrase, suggesting the expression was dependent on the cell growth phrase (Fig. 8B). After 72 h, the *qsdH* expression was not detected in this condition, and at the same time the signal molecule, 3OC8HSL, was accumulated. Therefore, the AHLs accumulated in *P. byunsanensis* strain 1A01261 until the AHL-lactonse QsdH was disappeared in culture. The phenomenon indicated that the QsdH regulated the production of AHL in strain on log phrase of cell growth when strain was cultured in this condition. After 6 days, the AHLs might be degraded as pH changed in culture or utilized by cell lacking of nutrition in this phrase. The QsdH lactonase located in the RND-type inner membrane with the catalytic domain exposed to the surface may be a quorum-quenching mechanism of *P. byunsanensis* 1A01261 used to control the intracellular concentration of AHLs, and further to regulate the expression of relevant phenotypic genes. These findings illustrate the important roles of QsdH in microbe/microbe and pathogen/host interactions of *P. byunsanensis* 1A01261.

In conclusion, it was demonstrated that QsdH possesses interesting features with respect to both biological and biocatalyst functions. The localization of QsdH in the inner membrane with the catalytic domain oriented into the periplasm and its ability to hydrolyze AHLs suggest an important role for this lactonase *in vivo* and in *vitro*. The AHL-degrading activity of QsdH may perform useful functions that affect the quorum sensing-associated phenotypic characterization of genus *Pseudoalteromonas*. The attenuation of plant pathogenicity shows the possibility of biocontrol of gram-negative bacteria by the use of recombinant QsdH-overproducing microbes, indicating that *P. byunsanensis* might have an additional function, including the regulation of gram-negative pathogenic bacteria.
